# Immune cell–antibody interactions in health and disease

**DOI:** 10.1093/cei/uxac065

**Published:** 2022-06-26

**Authors:** Sophia N Karagiannis, James N Arnold

**Affiliations:** St. John’s Institute of Dermatology, School of Basic & Medical Biosciences, King’s College London, Guy’s Hospital, London, UK; Breast Cancer Now Research Unit, School of Cancer and Pharmaceutical Sciences, King’s College London, Guy’s Cancer Centre, London, UK; School of Cancer and Pharmaceutical Sciences, King’s College London, London, UK

**Keywords:** antibody isotypes, B cells, macrophages, eosinophils, dendritic cells, NK cells

## Abstract

The human immune system safeguards against pathogens through a multitude of cellular and molecular signals, involving different components of the innate and adaptive response. Contrastingly, autoimmune diseases, allergic conditions, and cancer evoke different aspects of these otherwise protective processes. Understanding the immunological hallmarks for each pathological setting is essential for improving prevention, diagnosis, prognosis, and treatment. The activatory states of immune effector cells, especially in relation to their direct or indirect interactions with antibodies, are important determinants of an efficient, protective response that results in target clearance and improved clinical outcomes. Dysregulation of effector cells and their functions alongside alternatively activated humoral immune responses may contribute to several chronic diseases including allergic inflammation, autoimmune disorders and cancer. This Review Series brings to the forefront several key activation and regulatory features of immune effector cells in different diseases including cancer, infection allergy, and autoimmunity. Specific attention is drawn on how antibodies can impact effector cell states, and their pro-inflammatory and immune protective functions. Articles in this Series discuss different effector cells and antibody isotypes in infection, inflammation, tolerance and cancer immune surveillance, covering basic and translational mechanisms, clinical and epidemiological insights into these immune responses. Understanding the critical attributes of immune cells, especially those needed to effectively engage antibodies, will undoubtedly help better exploit their potential for disease management and therapy.

Immune effector cells and their interactions with antibodies are integral components of natural immune surveillance and clearance of pathogens [1, 2]. However, these interactions also support the resolution phase of inflammation to limit the destruction of healthy tissue, prevent autoimmunity, and restore homeostasis. Adaptive immunity driven by B cells, T cells, and antibodies can efficiently target and neutralize pathogens, pathogen-infected cells, or cancerous cells [3–5]. Following disease resolution, alternatively activated immune cells and antibodies can contribute to immune regulation of effector cell activation and their cytotoxic functions. In different pathological settings, environmental, pathogen-, or cancer-derived stimuli may influence and dysregulate the interactions between immune cells and antibodies.

This issue of *Clinical and Experimental Immunology* features the Review Series entitled *Immune cell-antibody interactions in health and disease*. Here we focus on immune cells and their activatory states associated with infectious, autoimmune, allergic and malignant diseases, encompassing interactions between effector cells and antibodies. The aim of this Review Series is to explore our current understanding of these interactions, to provide new insight into how and where this interphase can be manipulated therapeutically for the rational design of superior antibody therapeutic approaches for improving outcomes in infectious and inflammatory diseases.

Innate effector cells such as natural killer (NK) cells and their Fc receptors (FcRs) are critical determinants of immune activation, and their contributions can be decisive for the therapeutic efficacy of antibodies. Peipp et al. [6] describe the roles of NK cells in cancer immune surveillance and cancer immunotherapy. They discuss NK cell functions in relation to expression of the low-affinity activating FcR, FcγRIIIa, and its interaction with monoclonal antibodies and bispecific antibodies directed to tumor-associated antigens (TAAs), while engaging NK cells via FcγRIIIa or other activating NK receptors such as p30 (NKp30), p46 (NKp46), and the NK group 2 member D (NKG2D). These interactions trigger lytic and pro-inflammatory immune mediator secretion, leading to antibody-dependent-cell-mediated cytotoxicity (ADCC), and enabling activation of other immune cell types and the complement cascade. Other strategies involve neutralizing soluble, shed MICA and MICB molecules which impair NK and T cell activities in the tumor microenvironment. Refining these approaches holds significant promise in overcoming tumor-associated immunosuppressive signals and for re-engaging the potent anti-tumor functions of NK cells.

Eosinophils are a fundamental cell in orchestrating the allergic response and fighting parasitic infection. However, as our understanding of these cells has deepened it has become apparent that eosinophils play a significant role in the immune response to a range of pathogens. Gaur et al [7]. discuss the regulatory roles of eosinophils in viral, bacterial, and fungal infections. The review discusses the ability of eosinophils to sense a range of pathogens through their expression or pattern recognition receptors (such as TLRs) and FcRs for detecting immunoglobulin (Ig)A, IgE, and IgG bound material, which elicit their degranulation and release of a variety of inflammatory mediators to support the clearance of infection. However, such responses can also damage healthy tissue and result in hyper-sensitivity reactions. The review considers the rationale for the use, and further development, of eosinophil-related drugs in the infection setting.

Macrophages are antibody effector cells and well-characterized players in the tumor microenvironment, known for supporting disease progression [8]. Osborn et al. [9] discuss tumor-associated macrophages (TAMs), their phenotypes, plasticity, and functions in their interactions with monoclonal antibodies in ovarian cancer. Several monoclonal antibodies targeting macrophages and their mechanisms which contribute to tumor progression have been developed. Antibodies targeting colony-stimulating factor 1 receptor and CCL2 have been designed to deplete TAMs or restrict their recruitment into tumors. Antibodies such as those targeting the IL-6 signaling axis can interfere with macrophage pro-tumor and metastatic functions and others directed against VEGF-A/VEGFR and Ang2/Tie2 pathways aim to counteract pro-angiogenic signals. The review also highlights two aspects of macrophage biology that can be modulated using therapeutic antibodies. Firstly, their plasticity, whereby agonistic monoclonal antibodies specific for the co-stimulatory molecule such as CD40 can be used to engage macrophage anti-tumor functions [10]. Secondly, through capitalizing on FcRs and FcR engagement with antibodies directed against TAAs to facilitate cytotoxic and phagocytotic killing of cancer cells; this can be further augmented by neutralizing “don’t eat me’ signals on tumor cells. The capacity of macrophages to effectively engage antibodies highlights therapeutic opportunities for harnessing these cells in the anti-tumor response.

Dendritic cells (DCs) are specialized antigen-presenting cells in the immune cell arsenal and are crucial for priming T cell responses for the clearance of pathogenic infection. Furthermore, DCs are also capable of raising anti-tumor T cells which have the potential to eradicate established cancers under the right conditions. Corogeanu and Diebold [11] discuss DCs and their direct and indirect engagement with therapeutic antibodies utilized for the treatment of cancer for the priming of anti-tumor immune responses. Direct methods include the use of antibodies targeting DC-associated receptors carrying TAAs (such as DEC-205, DC-SIGN and the mannose receptor, among others). Indirect strategies include the opsonization of TAAs on the tumor cell surface to facilitate antibody-dependent-cellular phagocytosis (ADCP). The review delves into the mechanism of action of these interactions and their influence on the modulation of the DC-mediated immune cascades in cancer, with the goal to improve successful outcomes from immune checkpoint inhibitors (ICI) and other immunotherapy approaches.

B cells are sources of antibodies that provide immune protection from invading pathogens but can also exert regulatory functions by autocrine or paracrine mechanisms. These functions can be potentiated either via ligand–receptor interactions or through cytokine or antibody production. The immune environment can influence B cell activation and functional attributes, and ultimately determine the overall anti- or pro-tumoral contributions of B cells in cancer. Two reviews in this Series focus on different critical functions of B cells. Human B cells can express five antibody classes (divided into nine subclasses or isotypes), each able to interact with complement components or with immune effector cells expressing FcRs, each specific for an Ig class. Each antibody class can exert distinct immune surveillance functions in different anatomic compartments, to confer host protection. Dysregulation of B cell class-switching mechanisms can result in preferential expression of certain antibody isotypes which can significantly influence engagement with cognate FcRs on immune effector cells. The consequences can be suboptimal clearance of pathogens, tolerance of malignant cell growth, or enhanced and prolonged activation of effector cells against self which can contribute to autoimmune diseases.

The review by Flores-Borja and Blair [12] discusses the mechanisms of induction of regulatory B cells (Bregs), their mechanisms of action and crosstalk with immune and cancer cells in tumors which support immune suppression and cancer growth. Well-known IL-10 but also TGF-β-producing Breg populations in autoimmune and inflammatory diseases have been reported in several cancers. The review considers the full array of effector molecules expressed by these cells and their roles in disease progression. These roles include the promotion of myeloid-derived suppressor cells and Treg induction, modulation of T cell recruitment, induction of angiogenesis, immune checkpoint molecule expression and pro-inflammatory and cytotoxic properties which are discussed. Bregs and their roles in different cancers appear to be heavily molded by the inflammatory conditions orchestrated by cancer cells and significant interactions with multiple immune cell types in different anatomical sites. The authors highlight the importance of considering Breg populations with respect to their location, local inflammation signals, and contributions to tumor-associated immune suppressive forces to develop more precise targeted approaches.

It is increasingly appreciated that antibodies produced by B cells as part of immune surveillance, maintaining homeostasis or in response to pathogenic or cancer challenges, may provide a level of protection from the growth of cancer [13]. The antigen specificity as well as the class or isotype of antibodies expressed by B cells can determine the potency and the quality of the humoral response and its immune protective effects [14]. In a systematic review, Monroy-Iglesias et al. [15] study the potential significance of antibodies as biomarkers to predict the risk of cancer. Different Ig isotypes and specificities such as antibodies recognizing TAAs, infectious antigens, and autoantigens are evaluated. The study reports that antibody isotypes may be linked to specific cancer risk, diagnosis, or outcomes. The findings point to associations of serum class-switched antibody families, IgG, IgA, and IgE levels, with reduced risk of specific cancer types. With regards to antibody specificity, analyses highlight links between seropositivity to specific pathogen antigens with enhanced risk of some cancer types which are linked with known infectious agents. Examples of these were human papilloma virus seropositivity with cervical cancer and hepatitis B virus reactive antibodies with the risk of hepatocellular carcinoma. Antibodies recognizing TAAs such as MUC1 and CA125 were associated with diagnosis of specific cancers. Together these findings point to the importance of antibody signatures as potential prognostic, co-diagnostic and predictive tools, perhaps in the context of certain malignant diseases.

Ongoing patient-focused, functional, epidemiological, and big data approaches will enhance the search for the key molecular and immunological features of immune effector cells to guide precision medicine. Future clinical breakthroughs will likely include the use of cellular immune patterns and antibodies as biomarkers and the design of novel antibodies as therapeutic tools, which should consider their interactions with immune cells.

## References

[1] Nimmerjahn F , Ravetch JV. Fcgamma receptors as regulators of immune responses. Nat Rev Immunol 2008, 8, 34–47. doi:10.1038/nri2206. 18064051

[2] Arnold JN , Wormald MR, Sim RB, Rudd PM, Dwek RA. The impact of glycosylation on the biological function and structure of human immunoglobulins. Annu Rev Immunol 2007, 25, 21–50. doi:10.1146/annurev.immunol.25.022106.141702. 17029568

[3] Marshall JS , Warrington R, Watson W, Kim HL. An introduction to immunology and immunopathology. Allergy Asthma Clin Immunol 2018, 14, 49. 30263032 10.1186/s13223-018-0278-1 PMC6156898

[4] Downs-Canner SM , Meier J, Vincent BG, Serody JS. B cell function in the tumor microenvironment. Annu Rev Immunol 2022, 40, 169–93. doi:10.1146/annurev-immunol-101220-015603. 35044794

[5] Waldman AD , Fritz JM, Lenardo MJ. A guide to cancer immunotherapy: from T cell basic science to clinical practice. Nat Rev Immunol 2020, 20, 651–68. doi:10.1038/s41577-020-0306-5. 32433532 PMC7238960

[6] Peipp M , Klausz K, Boje AS, Zeller T, Zielonka S, Kellner C. Immunotherapeutic targeting of activating natural killer cell receptors and their ligands in cancer. Clin Exp Immunol 2022. doi:10.1093/cei/uxac028. PMC9307233 35325068

[7] Gaur P , Zaffran I, George T, Alekberli FR, Ben-Zimra M, Levi-Schaffer F. The regulatory role of eosinophils in viral, bacterial, and fungal infections. Clin Exp Immunol 2022. doi:10.1093/cei/uxac038. PMC9307229 35467728

[8] Opzoomer JW , Anstee JE, Dean I, Hill EJ, Bouybayoune I, Caron J, et al. Macrophages orchestrate the expansion of a proangiogenic perivascular niche during cancer progression. Sci Adv 2021, 7, eabg9518. 34730997 10.1126/sciadv.abg9518 PMC8565907

[9] Osborn G , Stavraka C, Adams R, Sayasneh A, Ghosh S, Montes A, et al. Macrophages in ovarian cancer and their interactions with monoclonal antibody therapies. Clin Exp Immunol 2021. doi:10.1093/cei/uxab020. PMC9307234 35020853

[10] Yu X , Chan HTC, Fisher H, Penfold CA, Kim J, Inzhelevskaya T, et al. Isotype switching converts anti-CD40 antagonism to agonism to elicit potent antitumor activity. Cancer Cell 2020, 37, 850–866.e7. doi:10.1016/j.ccell.2020.04.013. 32442402 PMC7280789

[11] Corogeanu D , Diebold SS. Direct and indirect engagement of dendritic cell function by antibodies developed for cancer therapy. Clin Exp Immunol 2022. doi:10.1093/cei/uxac026. PMC9307232 35352109

[12] Flores-Borja F , Blair P. Mechanisms of induction of regulatory B cells in the tumour microenvironment and their contribution to immunosuppression and pro-tumour responses. Clin Exp Immunol 2022. doi:10.1093/cei/uxac029. PMC9307227 35350071

[13] Harris RJ , Cheung A, Ng JCF, Laddach R, Chenoweth AM, Crescioli S, et al. Tumor-infiltrating B lymphocyte profiling identifies IgG-biased, clonally expanded prognostic phenotypes in triple-negative breast cancer. Cancer Res 2021, 81, 4290–304. doi:10.1158/0008-5472.CAN-20-3773. 34224371 PMC7611538

[14] Karagiannis P , Gilbert AE, Josephs DH, Ali N, Dodev T, Saul L, et al. IgG4 subclass antibodies impair antitumor immunity in melanoma. J Clin Invest 2013, 123, 1457–74. doi:10.1172/JCI65579. 23454746 PMC3613918

[15] Monroy-Iglesias MJ , Crescioli S, Beckmann K, Le N, Karagiannis SN, Van Hemelrijck M, et al. Antibodies as biomarkers for cancer risk: a systematic review. Clin Exp Immunol 2022. doi:10.1093/cei/uxac030. PMC9307228 35380164

